# Variability of DNA Methylation within Schizophrenia Risk Loci across Subregions of Human Hippocampus

**DOI:** 10.3390/genes8050143

**Published:** 2017-05-15

**Authors:** W. Brad Ruzicka, Sivan Subburaju, Francine M. Benes

**Affiliations:** 1 Program in Structural and Molecular Neuroscience, McLean Hospital, Belmont, MA 02478, USA; ssubburaju@mclean.harvard.edu (S.S.); fbenes@mclean.harvard.edu (F.M.B.); 2 Department of Psychiatry, Harvard Medical School, Boston, MA 02478, USA; 3 Program in Neuroscience, Harvard Medical School, Boston, MA 02478, USA

**Keywords:** DNA methylation, HumanMethylation450, schizophrenia, bipolar disorder, postmortem human brain, epigenetics, hippocampus

## Abstract

Identification of 108 genomic regions significantly associated with schizophrenia risk by the Psychiatric Genomics Consortium was a milestone for the field, and much work is now focused on determining the mechanism of risk associated with each locus. Within these regions, we investigated variability of DNA methylation, a low-level cellular phenotype closely linked to genotype, in two highly similar cellular populations sampled from the human hippocampus, to draw inferences about the elaboration of genotype to phenotype within these loci enriched for schizophrenia risk. DNA methylation was assessed with the Illumina HumanMethylation450 BeadArray in tissue laser-microdissected from the stratum oriens of subfield CA1 or CA2/3, regions having unique connectivity with intrinsic and extrinsic fiber systems within the hippocampus. Samples consisted of postmortem human hippocampus tissue from eight schizophrenia patients, eight bipolar disorder patients, and eight healthy control subjects. Within these genomic regions, we observed far greater difference in methylation patterns between circuit locations within subjects than in a single subregion between subjects across diagnostic groups, demonstrating the complexity of genotype to phenotype elaboration across the diverse circuitry of the human brain.

## 1. Introduction

Recent years have brought exciting advances in our understanding of the genetic underpinnings of major mental illnesses with the progress of next-generation sequencing technologies and bioinformatic methods for analysis of the complex datasets they produce. The identification of 108 genome-wide significant schizophrenia associated genomic regions by the Psychiatric Genomics Consortium (PGC) in 2014 brought robust results from the field’s considerable investment into genome-wide association studies, although now with these regions in hand the quest to understand this debilitating disorder is of course still far from complete. These regions span more than 20 megabases of DNA (20877196 base pairs [1]), approximately two thirds the size of the entire human exome [2], and are estimated to confer only 3.4% of the variance in schizophrenia risk [3], leaving the bulk of disease liability still unexplained. 

Schizophrenia is a neurodevelopmental disorder that likely involves molecular pathologies throughout the vastly complex system of the human brain, shaped by genetic and environmental influences spanning the time from early development throughout the lifespan [4]. Genotype is the foundation from which the multitude of phenotypes expressed in any single cell (the epigenome, transcriptome, spliceosome, proteome, and so on) are elaborated through interaction with environmental [5] and stochastic factors [6] that are not as yet fully understood. In the central nervous system, phenotypes of single neurons or glia interact at the levels of cell types, individual regions and larger networks of regions to produce the phenotype of the entire individual, from which a complex syndrome such as schizophrenia or bipolar disorder may be displayed. In the context of the daunting complexity presented by these and other neuropsychiatric disorders whose pathology lies within the brain, it is pertinent to consider what the significance of disease-associated single nucleotide polymorphisms (SNPs) identified in peripheral tissues such as blood may be.

It is likely that epigenetic mechanisms play an important role during brain development and throughout the life cycle in defining the ways in which an individual’s genotype is expressed as unique molecular phenotypes within specific neuronal subtypes in subregions of the brain. With the goal of accessing this process occurring within the central nervous system, specific DNA methylation signatures have been suggested as biomarkers of disease in peripheral blood that may have utility in clinical diagnosis or treatment, but more work is needed before meaningful constructs emerge [7]. The utility of biomarkers of this type is complicated by the fact that DNA methylation patterns show more variance between separate tissues as well as subregions within the brain of a single individual than they do between a single subregion across multiple subjects. This has been reported for peripheral blood mononuclear cells as compared to multiple human brain regions [8,9].

Many groups are now working to investigate the mechanisms conferring risk associated with each PGC region, and DNA methylation is one potential mechanism of risk receiving a great deal of attention. Methylation quantitative trait loci (meQTLs) are genomic regions where the DNA sequence influences methylation of a region or regions of DNA in cis or in trans [10], and this effect may be specific to distinct cell or tissue types and developmental time periods. Two important recent studies [11,12] investigated postmortem human brain tissue across the lifespan to demonstrate that PGC regions are enriched for sites that are differentially methylated between pre- and post-natal life. Also, it was discovered that nearly 60% of PGC regions contain a significant meQTL, potentially contributing to those regions’ mechanisms of risk. These studies analyzed samples of homogenized whole cortex, and changes in DNA methylation across development were found to be related to shifting proportions of different cellular populations within the brain [11]. Also, while the first study investigated only dorsolateral prefrontal cortex in adult brain, the second compared three regions (prefrontal cortex, striatum, and cerebellum) and found that while most fetal meQTLs were stable, there were meQTLs that showed specificity with regard to time and brain region [12]. 

In light of these recent findings, we sought to investigate the variability of DNA methylation patterns within the 108 PGC regions across closely related circuit locations within the human hippocampus, and how this differs within and between individuals and diagnostic groups. The work described herein is a focused secondary analysis of a previously reported investigation of DNA methylation patterns in two distinct but highly similar cell populations sampled from hippocampal subregions CA1 and CA2/3 [13]. These subregions were sampled from a cohort of 24 postmortem human hippocampus tissue samples obtained from eight schizophrenia (SZ) patients, eight bipolar disorder (BD) patients, and eight healthy control (CON) subjects. Laser microdissection was used to dissect tissue specifically from the stratum oriens, the outermost cellular layer of the hippocampal formation containing exclusively GABAergic interneurons as well as supportive non-neuronal cell types [14], from subfields CA2/3 and CA1, the locations of the second and third synapses within the trisynaptic pathway, respectively. DNA methylation was assessed in each of the 48 samples using the Illumina HumanMethylation450 BeadArray, and the analysis was restricted to the 5476 probes assessing cytosine residues found within the 108 PGC regions. Consistent with prior studies [8], our results demonstrate greater differences in methylation patterns between circuit locations within individuals than between single circuit locations across diagnostic groups. These data reinforce the difficulty of extrapolating genetic signals to phenotypic events across distinct tissues, and showcase one aspect of heterogeneity within the brain that cannot be appreciated through measurements performed in homogenized whole brain structures or in peripheral tissues.

## 2. Materials and Methods

### 2.1. Postmortem Human Hippocampus Tissue Samples

This work investigated a previously described [13] cohort containing postmortem human hippocampus tissue samples from 24 individuals, eight with schizophrenia, eight with bipolar disorder, and eight healthy controls, obtained from the Harvard Brain Tissue Resource Center (Table 1). Groups were matched for gender, age, postmortem interval, and tissue pH. All cases were obtained through family referral, not through the office of a medical examiner, and cases with documented history of serious drug abuse were excluded.

On arrival to the center, the fresh brains were dissected and the hippocampus was removed and sectioned. Tissue blocks used in this study were taken from the level of the pulvinar thalami along the rostro-caudal axis of the hippocampus. Upon removal, tissue blocks were fixed in ice-cold 0.1% formalin in 0.1 M phosphate buffer (pH 7.2) for 90 min before cryoprotection with 30% sucrose in phosphate buffer overnight and then stored at −80 °C.

### 2.2. Sample Processing and DNA Methylation Measurement

Tissue blocks were embedded in Optimal Cutting Temperature compound (Sakura Finetek, Torrance, CA, USA) and cut to 30 µm at 20 °C in a Microm HM560 cryostat. Sections were mounted on polyethylene terephthalate frame slides (Leica, Wetzlar, Germany), Nissl stained and dehydrated with ascending concentrations of ethanol. Sections were dried and stratum oriens tissue was dissected from CA1 and from CA2/3 using a Leica LMD6500 laser microdissection system. DNA was extracted from each sample using the QIAamp DNA Micro Kit (Qiagen, Hilden, Germany) and stored at −80 °C.

500 ng of genomic DNA was bisulfite modified using the EZ DNA Methylation Kit (Zymo Research, Irvine, CA, USA) using modified parameters recommended by Illumina, and modified DNA was assessed using the Illumina HumanMethylation450 BeadArray (Illumina, San Diego, CA, USA) as per the manufacturer’s protocol.

### 2.3. Data Analysis

Data was analyzed within R version 3.3.2. (R Foundation, Vienna, Austria). Raw intensity idat files were read into the minfi Bioconductor package [15] and whole-array data was normalized by stratified quantile normalization. No samples were identified as outliers and all samples were included in the analysis. M values (logit transformed β values which represent methylation ratios between 0 and 1) were extracted and then the dataset was restricted to the 5476 probes assessing cytosine residues within the original 128 genomic regions reported by the PGC [1]. Differentially methylated positions (DMPositions) were identified with false detection rate (FDR) < 0.05 using linear regression with the DMRCate Bioconductor package’s “myannotation” function [16] controlling for case (in comparisons across regions within individual subjects), age, and postmortem interval (PMI). Differentially methylated regions (DMRegions) were identified with Stouffer corrected *p* value < 0.05 with the “dmrcate” function within the DMRCate Bioconductor package, again with case (in comparisons across regions within individual subjects), age, and PMI included as covariates.

## 3. Results

### 3.1. Differentially Methylated Positions

The Illumina HumanMethylation450 BeadArray was used to investigate diagnosis associated changes in DNA methylation within the 108 PGC schizophrenia associated genomic regions in postmortem human hippocampus tissue from schizophrenia, bipolar disorder, and healthy control subjects. Investigation of the data for DMPositions identified only a single significant diagnosis associate difference among all four comparisons (5476 probes compared between SZ CA1 and CON CA1, SZ CA2/3 and CON CA2/3, BD CA1 and CON CA1, and BD CA2/3 and CON CA2/3). In the SZ CA2/3 vs. CON CA2/3 comparison, probe cg07925823 within the first exon of the SLC7A6 gene on chromosome 16 was significantly different between groups with FDR = 6.2 × 10^−3^. 

**Figure 1:**
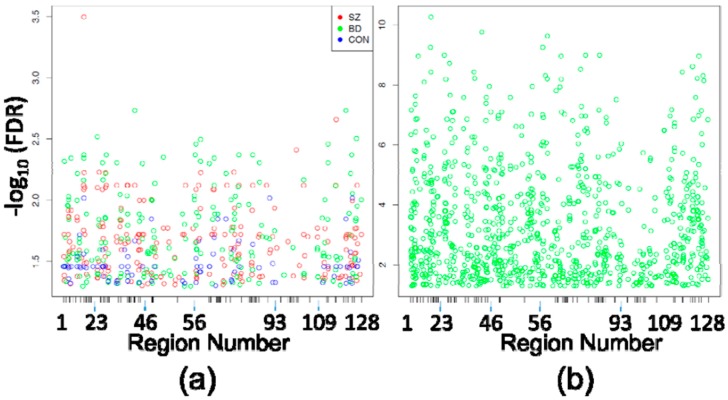
Differentially Methylated Positions (DMPositions) in CA1 vs. CA3. (**a**) Shown are the DMPositions identified within each eight-case diagnostic subgroup comparison (210 in SZ CA1 vs. SZ CA2/3, 201 in BD CA1 vs. BD CA2/3, 86 in CON CA1 vs. CON CA2/3) with genomic location within the space corresponding to the 5476 HM450 probes assessing sites within the original 128 PGC regions (5476 probes within those regions ordered from chromosome 1 to chromosome X, p to q) depicted on the X axis, and the −log10(FDR) on the Y axis. X-axis ticks mark the boundaries between PGC regions. As seen by the loose vertical columns, the DMPositions from separate diagnostic subgroups have a high degree of overlap, which is detailed in Table 2; (**b**) The 966 DMPositions identified in the pooled analysis of all 24 CA1 samples vs. all 24 CA2/3 samples are depicted within the same X and Y axes as Figure 1a.

Assessment of differences between circuit locations, on the other hand, identified 497 DMPositions among all three comparisons (5476 probes compared between SZ CA1 and SZ CA2/3, BD CA1 and BD CA2/3, and CON CA1 and CON CA2/3) (Figure 1a, Table 2 and Supplementary Materials file), and 966 DMPositions between CA1 and CA2/3 by pooling all 24 cases without consideration of diagnostic categories (Figure 1b and Supplementary Materials file). In Figure 1a, it is apparent that DMPositions within and between diagnostic groups cluster around certain genomic loci, generating the loosely columnar appearance of the plot. More than 50% of individual probes corresponding to circuit location DMPositions were shared between separate diagnostic subgroup comparisons, and 95% to 99% of individual DMPositions identified in circuit location comparisons within diagnostic subgroups were also identified as DMPositions in the pooled analysis (Table 2).

### 3.2. Differentially Methylated Regions

**Figure 2:**
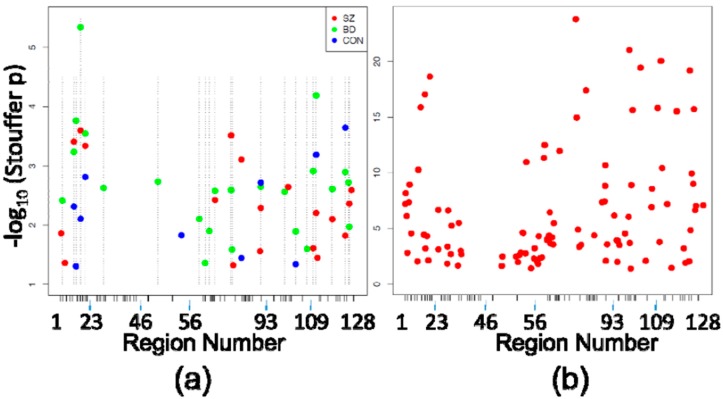
Differentially Methylated Regions (DMRegions). (**a**) Similar to Figure 1a, shown are the DMRegions identified within each eight-case diagnostic subgroup comparison (19 in SZ CA1 vs. SZ CA2/3, 23 in BD CA1 vs. BD CA2/3, 10 in CON CA1 vs. CON CA2/3), with the X axis again depicting the space corresponding to the 5476 HM450 probes assessing sites within the 128 PGC regions, and the Y axis depicting the −log10 (Stouffer corrected *p*-value) of each DMRegion. X-axis ticks mark the boundaries between PGC regions. Dashed vertical lines indicate the locations of BD-associated DMRegions (the most abundant) to demonstrate the scarcity of DMRegions that are not shared across comparisons within the separate diagnostic subgroups; (**b**) The 103 DMRegions identified in comparison of all 24 CA1 samples vs. all 24 CA2/3 samples are plotted against the same axes as Figure 2a.

Investigation of DMRegions yielded similar results, with zero significant DMRegions detected in any of the four between-diagnosis comparisons described above, and multiple DMRegions between each circuit location within diagnostic subgroups. Figure 2a shows the 19 significant DMRegions between SZ CA1 and SZ CA2/3, 23 DMRegions between BD CA1 and BD CA2/3, and ten DMRegions between CON CA1 and CON CA2/3, and shown in Figure 2b are the 103 significant DMRegions between CA1 and CA2/3 in all cases, pooled without consideration of diagnosis. As with DMPositions, there is a large amount of overlap of circuit location of DMRegions between the diagnostic subgroups (Table 3), with approximately half as many DMRegions observed in the control subjects as there were in each of the patient groups.

## 4. Discussion

As our understanding of the complex structure of genetic risk for schizophrenia advances, an important next step in understanding this disease will be the difficult task of mapping that structure onto the complex neural circuitry of the human brain. Even in unicellular organisms, the relation between genotype and phenotype is highly complicated, as a given mutation informs phenotype through a set of modifiers within the genetic background that are specific to an individual organism [17,18]. Considering this single-cell complexity in concert with the extraordinary diversity of neuronal and glial cell types and circuits within the human brain, a robust understanding of how a genotype is elaborated into the unique phenotypes within the many cells that comprise the complex circuits found within individual cortical layers, regions, and subregions presents a formidable challenge both in health and in disease states such as schizophrenia.

Within the 108 PGC schizophrenia-associated genomic regions, we observed only a single DMPosition in all four of the diagnostic comparisons, as opposed to 497 DMPositions in the three circuit location-based comparisons. Similarly, there were no DMRegions identified in the four diagnostic comparisons, and a total of 52 DMRegions across all three circuit location comparisons. This finding of greater differences in DNA methylation patterns between brain regions from the same individual than exist within a single brain region across separate subjects is in keeping with previous work [8], and to our knowledge these data represent the most subtle comparison yet made. Interestingly, a more recent study [19] drew the exact opposite conclusion, finding that methylation differs more between individuals than between brain regions (with the exception of cerebellum compared to cerebral cortical regions). While the cause of this disagreement is unknown, multiple technical differences between the studies (Methylated DNA Immunoprecipitation Sequencing vs. MBD Affinity Purification Sequencing, distinct brain regions sampled, and different analytic techniques) may have contributed.

These data are consistent with another recently published study that investigated schizophrenia-associated DNA methylation changes across four distinct regions of the human brain in homogenized whole cortex, accounting for estimates of neuronal/glial proportions in each sample [20]. The prior study found multiple DMPositions and DMRegions between schizophrenia and control subjects, and while many were shared across brain regions, the majority of these changes were significant in only a subset of regions, with the cerebellum being the most distinct region assayed. In alignment with our findings, the prior study found no enrichment of schizophrenia-associated DMPositions or DMRegions within the 108 PGC regions. Very interestingly, this prior work found the association of DNA methylation with a schizophrenia polygenic risk score based on the PGC GWAS results to be much more consistent across brain regions than was the association of DNA methylation with a formal diagnosis of schizophrenia. 

As detailed in Table 2, the average methylation change for all DMPs observed in our analysis was 4.5%, a value in keeping with many methylation change findings published from similar investigations [12,20,21]. The potential functional impact of a change of this magnitude within a complex heterogenous system such as the human hippocampus is difficult to predict and likely dependent on a great many factors, including the specific genomic context of each cytosine residue in question. While our laser-microdissected samples contain a more homogenous population of cells than samples of homogenized brain structures, they are still composed of multiple distinct cell types (i.e., glia and multiple GABAergic interneuronal cell types). Methylation changes are certainly not evenly distributed among all cells assessed by our measurements, and as such some populations are likely to host greater methylation differences between circuit locations and some populations lesser differences. As technologies continue to allow for the assessment of increasingly homogenous neuronal populations, effect sizes of identified disease and circuit location-associated methylation changes will likely increase. Another point in consideration of the functional importance of these differences is that the field is currently elucidating multiple roles for DNA methylation beyond the direct regulation of gene expression, including the regulation of alternate promoter usage, alternative splicing events, and support of transcriptional elongation [22]. 

While the samples employed here are far more specific than homogenized whole cortex, which is the most common method used to investigate human brain tissue, it is still essential to account for possible shifts in cellular populations between samples. The stratum oriens of the hippocampus contains exclusively GABAergic interneurons in addition to glial cells [14,23]. There exist tools to estimate the proportion of neuronal and non-neuronal cells within a sample based on its DNA methylation profile (i.e., the estimateCellCounts function within the minfi Bioconductor package [15]), however, these tools require a preexisting DNA methylation dataset from fluorescence-activated cell sorted (FACS) tissue. No such dataset is available for stratum oriens tissue from the human hippocampus, and available datasets from FACS sorted homogenized whole cortex are not applicable to our data. Previous work in our lab assessing gene expression profiles in equivalently microdissected stratum oriens tissue from CA1 and CA3 in a non-overlapping cohort of postmortem human hippocampus [24] found no significant difference between circuit locations in the expression of the glial specific mRNA marker GFAP (encoding glial fibrilary acidic protein) or the neuron specific mRNA ENO2 (encoding enolase 2, also known as neuron specific enolase) as normalized to the expression of the housekeeping gene GAPDH (encoding glyceraldehyde 3-phosphate dehydrogenase). Based on these findings, we are confident that the reported DNA methylation changes between the stratum oriens of CA1 and CA3 are not due to different proportions of neurons and glia present at these circuit locations, but instead depict distinct methylation profiles within phenotypically equivalent cellular populations.

The present analyses included a relatively small cohort, and greater numbers may have identified additional DMPositions or DMRegions, but the general observation of more between circuit location differences than between-subject differences is still valid. Additionally, the lack of genotyping data for the subjects precluded searching for meQTLs within the PGC regions in our cohort, as others have done previously [11,12]. One further potential limitation of this study is our inability to investigate CpG sites within the PGC regions not targeted by probes on the HM450 BeadArray. Indeed, there are 25 PGC regions associated with zero HM450 probes, and another 14 regions associated with only a single probe. Again, these shortcomings do not undermine conclusions regarding the within vs. between-subject comparisons described above.

While patients within the SZ and BD groups were treated with psychotropic medications [13], we argue that medication is not driving the observed changes in DNA methylation for two reasons. First, we observed more DNA methylation differences within between-circuit comparisons, where medication exposure is perfectly balanced as the same individuals are present in the CA1 group and the CA2/3 group, as opposed to comparisons across diagnostic groups of separate individuals. Second, there is a large degree of overlap in DMPositions and DMRegions found in the three diagnostic groups, including the non-medication exposed control group. We cannot exclude the possibility that medication exposure is causal of the twofold greater numbers of DMPositions and DMRegions observed in the SZ and BD groups as compared to the CON group, and this greater difference across circuit locations in psychotic disorders as a result of either the disease process itself or its pharmacologic treatment is an interesting finding requiring further study.

## 5. Conclusions

Even as basic a molecular phenotype as DNA methylation patterns shows variation across closely related cellular populations such as those found within a single cellular layer in distinct subfields of the human hippocampus within individual subjects. The 108 PGC schizophrenia-associated genomic regions represent a subset of the genome where inter-individual genetic variation is likely to be especially relevant to an individual’s risk for schizophrenia. Our finding of greater differences in DNA methylation of these regions between circuit locations within the identical genetic background of the same individuals as compared to the differences between genetically diverse groups of patients and controls illustrates the difficulties of determining the impact of a given genotype on the complex phenotypes encompassed by a neuropsychiatric illness such as schizophrenia. Understanding the influence of genetic variants on the complex landscape of schizophrenia risk within the many distinct cell types, circuits, tissue microenvironments, and time points relevant to this disease is the next necessary step in this ongoing and grand challenge.

## Figures and Tables

**Table 1:** Summary of demographic measures within the cohort.

Diagnosis	*n*	Gender	Age	PMI	pH
CON	8	4M/4F	64.1 ± 14.2	22.4 ± 5.7	6.4 ± 0.2
SZ	8	4M/4F	67.9 ± 17.3	26.4 ± 10.3	6.4 ± 0.2
BD	8	3M/5F	68.3 ± 11.7	25.0 ± 6.7	6.3 ± 0.2
*p*		0.86	0.82	0.58	0.46

Demographic variables averaged within each diagnostic subgroup of the cohort are listed, with *p* values for each measure generated by ANOVA provided in the final row. Full details for individual subjects are available in a previous description of this cohort [13]. CON: control; SZ: schizophrenia; BD: bipolar disorder; PMI: postmortem interval.

**Table 2:** Distribution and overlap of differentially methylated positions among diagnosis and circuit location comparisons.

Comparison	# of DMPs	Mean Delta Beta	Mean FDR
SZ CA1 vs. SZ CA2/3	210	0.064	0.025
BD CA1 vs. BD CA2/3	201	0.063	0.023
CON CA1 vs. CON CA2/3	86	0.075	0.034
Pooled Cases CA1 vs. CA2/3	966	0.035	0.0087
SZ CA2/3 vs. CON CA2/3	1	0.035	0.0062
SZ CA1 vs. CON CA1	0	NA	NA
BD CA2/3 vs. CON CA2/3	0	NA	NA
BD CA1 vs. CON CA1	0	NA	NA
**Comparison**	**# of DMPs**	**% of DMPs**	
SZ vs. BD	107	53.23	
SZ vs. CON	53	61.63	
BD vs. CON	59	68.6	
All 3 Groups	46	53.49	
SZ vs. Pooled	199	94.76	
BD vs. Pooled	195	97.01	
CON vs. Pooled	85	98.83	
All vs. Pooled	46	100	

At the top are listed the number of DMPositions identified in each circuit location (i.e., SZ CA1 vs. SZ CA2/3) or diagnostic group (i.e., SZ CA1 vs. CON CA1) comparison, and the mean delta beta and mean FDR for all DMPs in that comparison. At the bottom is the overlap of specific CpG sites among the various comparisons, with # of DMPs indicating the number of DMPositions that are common to the two listed comparisons (i.e., SZ vs. BD indicates there are 107 DMPositions common to both the SZ CA1 vs. SZ CA2/3 and the BD CA1 vs. BD CA2/3 comparisons). All three groups indicate there are 46 DMPositions common to all three within diagnostic subgroup comparisons. % of DMPs lists the percentage of DMPositions from the comparison with the smaller number of DMPositions that are common to both indicated comparisons (i.e., for SZ vs. BD 107 common DMPositions/201 BD CA1 vs. BD CA2/3 DMPositions = 53.23%). DMP: differentially methylated position; FDR: false detection rate.

**Table 3:** Distribution and overlap of differentially methylated regions among circuit location comparisons.

Chr	Start	End	Description	SZ	BD	CON	Pooled
chr01	2381300	2381623	26 kb upstream of PLCH2	13			28
chr01	2391242	2391347	16 kb upstream of PLCH2		16		
chr01	8484005	8484417	RERE intron 12	18			24
chr01	150123038	150123734	PLEKH01 spans exon 2	3	5	5	21
chr01	150229144	150230345	CA14 TSS		3	10	9
chr01	243584669	243584861	SDCCAG8 intron #15	1	1	6	8
chr02	72357938	72358024	CYP26B1 3′ end	4	4	3	6
chr03	2553086	2553187	CNTN4 intron 3		11		39
chr06	28499677	28499825	GPX5 exon 3 3′ boundary		8		83
chr07	1983170	1983503	MADL1 intron 16			7	18
chr07	2143508	2144767	MADL1 intron 12		17		17
chr07	86413439	86414302	GRM3 intron 2		23		40
chr07	104909431	104909815	SRPK2 exon 3 5′ boundary		19		45
chr10	18689036	18689503	CACNB2 exon 5 5′ boundary	8	14		16
chr11	46365894	46367100	DGKZ exon 1	2	13		1
chr11	46383032	46383209	DGKZ exon 4		22		14
chr11	46401423	46401447	DGKZ exon 32	19			47
chr11	57414402	57414908	YPEL4 spans exon 2	5		8	7
chr12	57589254	57589740	LRP1 exon 53	16			29
chr12	57597137	57597238	LRP1 exon 70 5′ boundary	10	10	4	19
chr14	104171260	104172224	XRCC3 intron 6		15		2
chr15	40599681	40600635	PLCB2 TSS	6			12
chr15	91426668	91427884	Overlaps FURIN 3′ end & FES TSS		20	9	4
chr16	30124804	30124904	GDPD3 3′ end		21		36
chr16	67918485	67919362	NRN1L TSS	15	6		10
chr16	67977866	67978450	SLC12A4 exon 24	11	2	2	3
chr16	68000764	68001415	CLC12A4 exon 2 5′ boundary	17			20
chr17	18011514	18012134	MYO15A TSS	12	12		13
chr20	37464180	37464594	PPP1R16B exon 2	14	7	1	5
chr22	41613693	41613790	L3MBTL2 intron 5		9		23
chr22	41636942	41637617	CHADL TSS	9	18		11
chr22	42347907	42348061	LINC00634 TSS	7			35

Listed are the locations of all DMRegions identified in the three within-diagnosis comparisons of CA1 and CA2/3. Numbers depict the rank order of that DMRegion in the indicated comparison, and missing numbers indicate that the associated region was not detected as significantly differentially methylated in that comparison.

## Supplementary Materials

The following are available online at www.mdpi.com/2073-4425/8/5/143/s1.
